# Assessing the Environmental and Socio-Economic Impacts of Artisanal Gold Mining on the Livelihoods of Communities in the Tarkwa Nsuaem Municipality in Ghana

**DOI:** 10.3390/ijerph13020160

**Published:** 2016-01-26

**Authors:** Samuel Obiri, Precious A. D. Mattah, Memuna M. Mattah, Frederick A. Armah, Shiloh Osae, Sam Adu-kumi, Philip O. Yeboah

**Affiliations:** 1 Department of Nuclear Sciences and Applications, School of Nuclear and Allied Sciences, Ghana Atomic Energy Commission, P.O. Box AE 1, Atomic, Accra, Ghana; s.osae@gaecgh.org (S.O.); poyeboah47@yahoo.co.uk (P.O.Y.); 2 Centre for Environmental Impact Analysis, P.O. Box AD 738, Cape Coast, Ghana; 3 Directorate Academic Planning and Quality Assurance Unit, University of Cape Coast, Cape Coast, Ghana; pmattah@ucc.edu.gh or pmattah@yahoo.com; 4 Department of Environmental and Development Studies, Central University College, P.O. Box 2305, Tema, Ghana; mbmattah@yahoo.com or mmattah@central.edu.gh; 5 Department of Environmental Science, University of Cape Coast, Cape Coast, Ghana; farmah@ucc.edu.gh; 6 Environmental Protection Agency, P.O. Box M 326, Accra, Ghana; adukumisam@yahoo.com

**Keywords:** heavy metal, mining impact, integrated assessment, Tarkwa Municipality, risk perception, water quality, social issues, livelihood and economic issues, artisanal gold mining

## Abstract

Gold mining has played an important role in Ghana’s economy, however the negative environmental and socio-economic effects on the host communities associated with gold mining have overshadowed these economic gains. It is within this context that this paper assessed in an integrated manner the environmental and socio-economic impacts of artisanal gold mining in the Tarkwa Nsuaem Municipality from a natural and social science perspective. The natural science group collected 200 random samples on bi-weekly basis between January to October 2013 from water bodies in the study area for analysis in line with methods outlined by the American Water Works Association, while the social science team interviewed 250 residents randomly selected for interviews on socio-economic issues associated with mining. Data from the socio-economic survey was analyzed using logistic regression with SPSS version 17. The results of the natural science investigation revealed that the levels of heavy metals in water samples from the study area in most cases exceeded GS 175-1/WHO permissible guideline values, which are in tandem with the results of inhabitants’ perceptions of water quality survey (as 83% of the respondents are of the view that water bodies in the study area are polluted). This calls for cost-benefits analysis of mining before new mining leases are granted by the relevant authorities.

## 1. Introduction

The notion of sustainable development has given rise to various visions of the future of the world, possible trade-offs and of externalities [1]. Although sustainable development requires the integration of the economic, environmental and social dimensions of development, the economic considerations often override the environmental and social considerations in most developing countries, including Ghana. Many of the social elements of sustainable development can be cast in the light of socio-economic considerations as links between the economic, environmental and social dimensions. When dealing with the social dimensions of gold mining, the ultimate goal should be on identifying the ways to maximize the positive effects of mining on the lives of people while minimizing the negative effects. These effects should reflect the impact of mining on the present generations as well as future generations of miners and their families. It must also be noted that the social dimension is subjective, qualitative, difficult to measure and perceived differently by the various players and stakeholders within the gold mining sector. Furthermore, environmental considerations are linked to discussions on health and safety, settlements, and the impact of gold mining on subsistence lifestyles.

Ghana’s social history is inextricably linked to natural resources. Gold mining in Ghana has played a central role in the social, economic and political life of the nation for over 2000 years [2]. However, in recent times, a lot of Ghanaians have become aware of the deleterious effects of gold mining while at the same time recognizing it’s important role in the national economy [3,4]. Several researchers have documented the environmental and socio-economic impacts of gold mining in other countries [5,6,7,8]. However, due to research specialization and discipline-specific worldviews, seldom does research integrate the natural and social dimensions of the impacts of gold mining in a single paper [8,9].

For instance, in Ghana most natural scientific studies on impacts of gold mining on the environment have concentrated exclusively on measuring the levels of toxic chemicals such as arsenic, cyanide, cadmium, lead and mercury in water, soil and food crops grown in mining communities [4,7]; while other studies have focused on assessing cancer and non-cancer human health risks to residents of mining communities associated with toxic chemicals via oral and dermal contact with the toxic chemicals in polluted water [7,10,11,12]. On the other hand, the social-scientific studies have focused on assessing the socio-economic status of mining-dependent areas and on the socio-economic costs or effects of mine closures and their views on future development of mines [13,14]. There appears to be very little overlap between the two fields of research on gold mining, and there is a paucity of research on the cumulative and integrated impacts of artisanal gold mining on host communities. This study employs integrated assessment framework of artisanal and small-scale gold mining in Ghana and seeks to develop a more holistic understanding of the risks and benefits of gold mining by evaluating heavy metal contamination in waters adjacent to mining areas coupled with social assessment of residents’ perceptions about water quality and water pollution as well as livelihood implications of artisanal gold mining the municipality [8,9,14].

The underlying hypothesis for this study is that irrespective of a person’s educational background they have some knowledge of environmental pollution and can use indigenous knowledge such as color, taste of the drinking water to tell whether the water is polluted or not. The objectives of the present study are:

1. To investigate and evaluate the water pollution in artisanal gold mining impacted communities in Tarkwa Nsuaem Municipality.
2. To assess the perceptions of inhabitants related to the poor water quality of the river and their drinking water.
3. It also seeks to assess the livelihood implications of residents due to artisanal small-scale gold mining in the municipality.

## 2. Materials and Methods

### 2.1. Study Area

**Figure 1:**
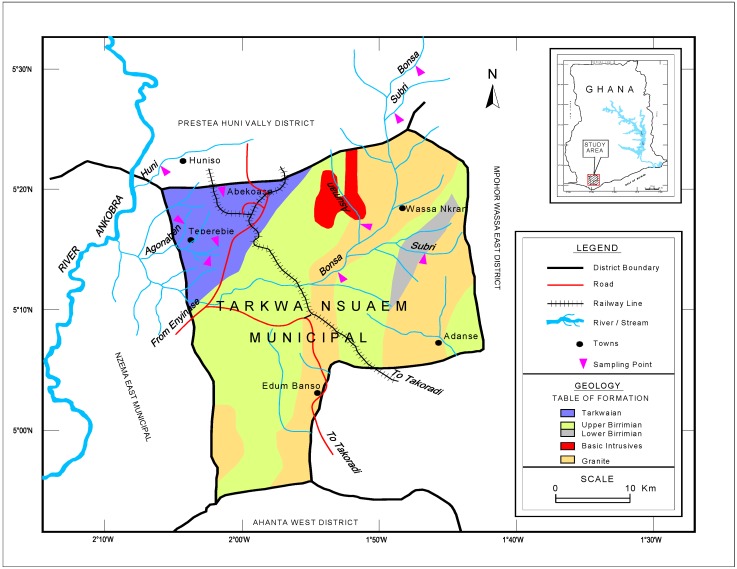
Map of the study area.

The Tarkwa Nsuaem Municipality is one of the twenty-two districts in the Western region of Ghana. It has a total land surface area of 2354 square kilometers, which lay between latitude 40°50′ N and 50°40′ and longitude 10°45′ and 2° W (Figure 1). The study area produces an estimated 35% of Ghana’s gold output [15]. The municipality has evergreen mountain ranges, which present an appealing aesthetic scenery for people living in the area. Unfortunately, these ridges are the main gold-containing areas and are targeted for open cast mining, so they have undergone tremendous mining-related development in recent decades [15]. The Tarkwa Nsuaem municipality has three forest reserves, which are the Bonsa, Ekumfi and Neung reserves, covering 440.15 km^2^ of land surface area and representing about 10% of the country’s close forest that is rich in flora and fauna [15]. The population of Tarkwa Nsuaem Municipality, according to the 2010 Population and Housing Census, is 90,477, with relatively more males (51.6%) than females (48.4%). The population of the Municipality is youthful, with about two-fifths (38.1%) aged below 15 years and a smaller proportion (4.4%) of elderly persons (aged 60 years and older). The total age dependency ratio for the Municipality is 69.6 with the females ratio (72.6) being higher than that of males (67.1). Also, most of the residents live in rural areas where access to basic amenities such as potable water (treated pipe-borne water) and education are virtually non-existent [15].

The majority of the people in the area, especially the rural women, scout for oil palm, snails, herbs, spices and firewood from the forest as their main source of income. The livelihoods of community members and much of the informal economy in the study area are heavily dependent on natural resources extracted from the surrounding forested area.

### 2.2. The Geology of the Study Area

The gold bearing ores in Ghana are obtained from the Birimian and the Tarkwaian rock system as shown in Figure 1 above [16]. The gold found in the three main types of auriferous deposits are:

- Reef, vein or lode-types gold deposits, defined as gold deposits found in quartz-veins but in a broader sense this refers to any gold bearing lode or dyke. They are usually found in the Birimian rock systems.
- Auriferous quartz-pebble conglomerates that occur as intrusive veins in phyllites and greenstones within the Birimian and the Tarkwaian rock system. The veins are found in two main places (1) near or at the contact of the Lower and Upper Birimain rocks; and (2) the reefs within the Birimian rocks.

The gold is trapped or entangled in the crystal structure of the sulphides and the oxides bearing rocks together with other mineral ores such as sphalerite (ZnS), galena (PbS), chalcopyrite (CuFeS_2_), enargite (Cu_3_AsS_4_) and other radioactive materials [16,17]. Weathering of waste rocks, as well as improper discharge of mining tailings or mine process solution releases toxic chemicals into the environment which may impact residents of mining communities and mine workers [3].

Exposure to the abovenamed toxic chemicals in the spilled mine tailings via oral, dermal and inhalation can be toxic human beings as they produce multiple adverse health effects at very low concentrations, and because of their ability to accumulate in the environment and human beings [18,19,20].

### 2.3. Sampling Techniques, Collection of Water Samples and Laboratory Analysis

Random sampling techniques were adopted in selecting 10 sampling points. Twenty samples were collected from January to October 2013 at each sampling point on a bi-weekly basis. In total, two hundred (200) water samples were collected from four streams, five rivers and one borehole into 1.5 L PET plastic containers rinsed with 1:1 HNO_3_ and distilled water. The samples were acidified with 2 mL nitric acid, stored in an ice-chest at a temperature below 4 °C and transported to the Council for Scientific and Industrial Research (CSIR) Water Research Institute’s laboratory for analysis. Analysis of samples was carried out within a week’s time. Samples yet to be analyzed were stored in a refrigerator at 4 °C.

### 2.4. Analysis of Mercury, Arsenic, Manganese, Lead and Cadmium

In the laboratory, the samples were filtered through 0045 μm Whatman filter paper. For the analysis of mercury, 5 mL of concentrated H_2_SO_4_ and 2.5 mL of concentrated HNO_3_ was added to filtrate (100 mL) and thoroughly shaken to get a homogeneous mixture. Five % (weight/weight or *w*/*w*) KMnO_4_ (15 mL) and 5% (*w*/*w*) potassium persulphate (8 mL) were added to the mixture which was heated at 95 °C for 2 h. The mixture was then allowed to cool to room temperature and 12% (*w*/*w*) hydroxylamine hydrochloride (6 mL) were added to the resulting solution to reduce the excess of permanganate. The digested solution was stored for analysis [21]. In the Hg determination, a blank solution containing all the reagents used in the digestion process excluding the sample was added to a carrier solution containing 3% (volume/volume or *v*/*v*) HCl and a reducing agent (1.1% mass/volume or *m*/*v* SnCl_2_ in 3% (*v*/*v*) HCl) to reduce all the mercury in the blank to mercury vapour which was determined by the cold vapour technique using a Shimadzu AAS model AA 6300 instrument. This was followed by the digested sample which was taken through the same treatment as the blank to generate Hg vapour which and analysed by cold vapour using the Shimadzu model AA 6300. The blank mercury concentration was automatically stored by the Shimadzu AAS model AA6300 and was subtracted automatically from the concentration to give the actual Hg concentration in the sample.

For the determination of cadmium, manganese, arsenic and lead, an acidified water sample (100 mL) was mixed with conc. HNO_3_ (5 mL). The mixture was heated until the mixture was reduced to about 20 mL on a hot plate. The digested samples were cooled to room temperature, filtered through a 0.45-μm Whatman filter paper and the final volume adjusted to 100 mL with double distilled water and stored for analysis [21]. The concentration of cadmium, manganese, arsenic and lead in the samples were determined using a flame Atomic Absorption Spectrophotometer. This was done after a blank solution prepared for the toxic chemical of interest using the same procedure outlined in the mercury determination above.

For the determination of As, 0.5 M HCl (5 mL) and 0.5% NaBH_4_ (5 mL) were added to a blank solution prepared from all the reagents used in the digestion of As in the water samples. The As in the blank was reduced to arsine gas in an arsine gas generator coupled to the Shimadzu flame AAS model AA 6300 instrument. The As concentration in the blank was stored automatically by the AAS. The As ions in the digested water samples were all reduced to arsine gas in the arsine gas generator following the addition 0.5 M HCl (5 mL) and 0.5% NaBH_4_ (5 mL). The arsine gas generator is coupled to the Shimadzu model AA 6300 flame atomic absorption spectrophotometer for the determination of As in the samples. The detection limits for Hg, As, Pb, Mn and Cd were all 10 μg*/*L, respectively.

### 2.5. Quality Control

Reproducibility and recovery studies were conducted using a certified reference material for water samples prepared by BDH Chemicals (London, UK) with a concentration of 10 μg/L each of As, Hg, Cd, Mn and Pb. The percentage of As, Hg, Cd, Mn and Pb recovered in the recovery studies ranged from 95% to 100% for As, Hg, Cd, Mn and Pb respectively. In the reproducibility studies, the percentage of As, Hg, Cd, Mn and Pb recovered by the Shimadzu AAS model AA 6300 ranged from 96.3% to 99.7% (standard error ± 0.005 to 0.560). The standard error was less than 1, suggesting that the method used in analyzing the samples were reproducible.

### 2.6. Social Survey

The household surveys were largely carried out by an enumeration of households within the study area which was carried out by resident enumerators (REs) and which lasted for six weeks. Although the eligibility criteria for selection of resident enumerators varied by community, the resident enumerators were typically individuals over the age of 21 years who were from, or near the respective enumeration areas and held a high school diploma, or had attained a higher level of education. The primary aim of the survey was to collect a representative sample of data from households in selected gold mining communities close to where the natural science team collected the water samples to assess their perception of gold mining impacts on water quality. The surveys involved interviewing 250 respondents aged between 21 years and older.

The questionnaire and the study protocol were approved by the Ghana Health Service Ethical Review Council—GHS-ERC: 07/5/13. For eligibility, only respondents who had resided continuously in the study area for at least 5 consecutive years and had agreed to the consent forms were recruited to be part of this study. Fundamental demographic information collected included, but was not limited to age, gender, education, employment, household income, and ethnicity. Also, information on geographic location, marital status, occupation, farm ownership, perceived environmental and health quality, involvement in gold mining and personal income were obtained.

Logistic regression analysis was applied to evaluate the outcome variable on perceived quality of drinking water source [22]. Respondents were asked “in your opinion, is the river which serves as your drinking water, polluted by gold mining activities?” In this analysis, the socio-economic and local environmental characteristics (see Table 1 below) represent the independent variables (predictors).

The research design implemented for the social survey was a quantitative, correlational design. Logistic regression analysis was performed to determine whether or not any of the independent variables influenced the dependent variable (drinking water source quality). A logistic regression model is: which is frequently used when the dependent variable is dichotomous [23]. Let *Y* be the dependent variable, which takes on values 1 (event) and 0 (nonevent). In this context, the event represents water source is perceived as polluted and nonevent represents water source is perceived as unpolluted. Further, let *p* denote the probability that an observation is an event, that is, *p* = *P*(*Y* = 1). The logistic regression models the log-odds of an event as a function of a linear combination of the intercept and slope parameters. With the obtained estimates, it can be shown that:

$$ln\left(\frac{p}{1-p}\right)=\alpha +\beta_{1}x_{1}+\beta_{2}x_{2}+...\beta_{k}x_{k}$$

$$p=\frac{\exp\{\alpha +\beta_{1}x_{1}+\beta_{2}x_{2}+...\beta_{k}x_{k}\}}{1+\alpha +\beta_{1}x_{1}+\beta_{2}x_{2}+...\beta_{k}x_{k}}$$

which gives the estimated probability that an observation is an event. Usually, when this probability is greater than 0.5, the observation is classified as event otherwise; it is classified as non-event [23].

**Table 1:** Socioeconomic and environmental variables of the perception study, showing questions asked and answers provided by the 250 households.

Socio-Economic Characteristics and Their Content	Category	% of the 250 Respondents
Independent variables		
*Demographic characteristics*		
Gender	Male *vs.* Female	51
Age	20–30 years	34
	31–40 years	18.5
	41–50 years	15
	51–60 years	17.5
	>60 years	15
Education	No formal education	39.6
	Junior High School	29
	Secondary	18
	Diploma	5
	University education	3.4
*Economic issues*		
Household income per day before mining	<$1 a day	52
	>$1 a day	48
Household income per day after advent of mining	<$1 a day	62
	>$1 a day	38
Current employment status	Unemployed	62
	Employed	22.5
	Retired	15.5
*Environmental issues attributed to mining (perception)*	Yes vrs No	67
Change in colour and smell of water bodies	Deteriorated	54
	Unchanged	34.5
	Improved	11.5
Drinking water quality	Bad	83
	Fair	12
	Good	5
Taste of drinking water		
Likely to have disease due to drinking water	Yes vrs No	86.5
Water pollution mentioned as a major environmental problem due to mining	Yes vrs No	83
Water pollution mentioned as a potential health risk	Yes vrs No	77
Surface mining pollutes water bodies	Yes vrs No	68
If surface mining operations stops water pollution in the community would cease	Yes vrs No	68
*Dependent variables*		
Surface mining deprives residents of mining communities their farmlands for the farm	Yes vrs No	90
Loss of farmlands means loss of livelihoods	Highly significant	87
	Not significant	13
Degree of mining induced pollution of water bodies	Highly polluted	86
	Not highly polluted	14
Degree of mining induced drinking water pollution	Polluted	84
	Not polluted	16
Stoppage of surface mining would improve the livelihoods of residents	Yes vrs No	83.5

## 3. Results and Discussion

### 3.1. Drinking Water Quality

The mean concentrations of heavy metals in water samples from water bodies in the study area are presented in Table 2 below. It was found out that 13% of the parameters measured in this study were found to be below the detection limit (10 μg/L respectively for As, Hg, Mn, Pb and Cd).

**Table 2:** Mean concentrations (μg/L) of heavy metals in filtered water samples from the study area. Data includes the minimum and maximum values from each sampling site. GSA-175A/WHO guideline for drinking water is shown for reference.

Sampling Point	As (μg/L)		Mn (μg/L)		Pb (μg/L)		Cd (μg/L)		Hg (μg/L)	
	Mean (SD)	Min–Max	Mean (SD)	Min–Max	Mean (SD)	Min–Max	Mean (SD)	Min–Max	Mean (SD)	Min–Max
River Achofe	**1246** (464.9)	15–2851	**490** (91.6)	124–580	**22** (4.80)	10–28	**21** (2.87)	17–32	**43** (14.9)	<10–64
River Agonaben	**89** (19.0)	14–120	234 (54.4)	36–300	**120** (47.4)	25–200	**396** (161.1)	19–974	**55** (23.3)	<10–94
River Adeyie	**184** (39.1)	43–248	222 (35.1)	100–313	**69.3** (26.8)	19–134	**321** (50.5)	115–341	**55** (26.2)	<10–120
River Bremansu	**59** (28.5)	<10–140	259 (51.6)	151–451	**165** (38.1)	13–214	**53.1** (25.4)	10–145	**98** (43.2)	<10–154
River Subri	**325** (100.8)	100–700	53 (24.7)	19–133	**20** (14.5)	<10–80	**231** (31.6)	134–329	**63** (17.1)	25–94
River Asuobenkasa	**112** (35.5)	43–248	191 (38.1)	79–313	**170** (35.8)	30–226	**391** (81.8)	47–420	**472** (107.9)	19–520
River Asuman	**15** (6.29)	10–40	26 (11.9)	<10–74	**122** (19.3)	40–127	**21** (6.39)	<10–44	**246** (96.5)	74 -480
Borehole at Abekoase	**20** (1.49)	18–24.5	103.2 (23.3)	50–147	**70** (21.2)	10–140	**137** (38.1)	10–241	**25** (7.38)	10–42
River Huni	**75.1** (19.4)	54–101	57.7 (24.7)	19–150	**66.9** (39.5)	10–221	**21** (21.2)	<10–109	**448** (91.4)	100–520
River Ateberebe	**133.9** (39.2)	125–300	31 (7.01)	12–60	**226** (56.2)	<10–329	**126** (55.8)	21–341	**83** (28.6)	<10–127
GS 175-1/WHO Guideline values	10		400		10		3		10	

Samples were collected from 10 sampling sites in TNMA on bi-weekly basis (over ten months; *i.e.*, January to October), these concentrations are the means; SD represents standard deviation. Bold figures exceeded the GS 175-1/WHO permissible guideline values [24].

**Figure 2:**
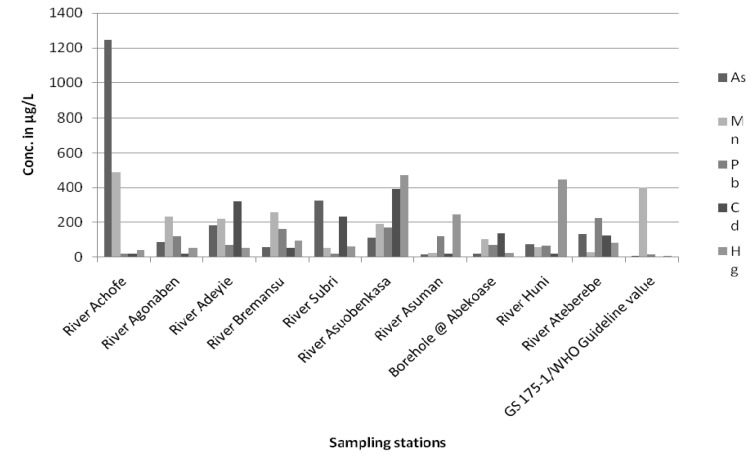
A graph comparing the levels of heavy metals in water samples from the study area with GS 175—1/WHO Permissible guideline values.

The mean concentrations of As ranged from 15 to 2451 μg/L. The mean As concentration in river Achofe was compared to the GS 175-1/WHO guideline, and it was found to have exceeded the required GS 17-1/WHO permissible guideline value [24], which is consistent with findings reported by [25]; the highest concentration of As (≥12,200 μg·L^−1^) measured in [24] corresponded to abandoned mine shafts in Konongo. This indicates that As pollution of the water bodies in the study area is extremely high. The high As contamination in the study area may be due to the dissolution of As in the arsenopyrite ores or indiscriminate discharge of mine effluents rich in As and other toxic chemicals into the environment [25]. Mean concentrations of As, Pb, Cd and Hg found in most of the water bodies sampled in this study exceeded GS 175-1/WHO guideline values; this assertion is consistent with findings made by [24]. A graphical comparison of the levels of the various chemicals in the water samples from the study area is presented in Figure 2 below. In the case of the As, Pb and Cd levels in water samples from the study area, it was found out that 100% of them exceeded the GS 175-1/WHO permissible guidelines [24]; while for Mn levels in the water samples, 10% of them were found to have exceeded GS 175-1/WHO permissible guidelines. Out of the 200 water samples analysed for Hg, it was found out that 100% of the samples exceeded the GS 175-1/WHO permissible guidelines values.

The source of these elevated levels of As, Cd, Mn, Pb and Hg in water samples from the study area is attributable to artisanal small-scale gold mining (ASGM). For instance, though As and Cd are naturally occurring metals, they are also associated with gold-bearing ores in the study area and are often found at elevated concentrations near gold mining sites [19,26,27,28]. In the Tarkwa area, elevated Cd concentrations may be the result of mining and processing of zinc and chalcophilic metals [19]. Some geologic formations, such as the Birimain and Tarkwanian rock systems found in the mining area of Tarkwa, contain high concentrations of Pb, as well as Mn [19]. Pb and Hg also derives from industrial discharges or mine drainage [19,28]. The weathering of ore tailings can lead to the leaching of heavy metals into other media such as water, soil, and sediment [7].

The pH values of water samples from the study area ranged from 5.59 to 7.45 with a mean value of 7.003, which shows that the water samples are neutral, *i.e.*, they are neither acidic nor basic (as shown in Table 3 below), as 60% of the samples had pH values of 6.43 out of the 200 samples from the study area.

**Table 3:** Descriptive statistics for physic-chemical parameter for water samples from the study area.

Parameter	Range	Mean	Standard Deviation	GS 175/1-WHO Guideline Values
pH	5.59–7.45	6.43	0.195	6.5–8.5
Turbidity	0.60–8.94	1.746	2.071	5
Colour	2.500–12.00	5.900	2.859	15
TDS	17.2–219.00	79.23	43.58	1000
Sodium	1.80–191.0	34.76	33.52	200
Magnesium	0.80–144.0	47.11	35.76	150
Calcium	0.50–112.0	27.68	25.76	200
Potassium	1.40–23.80	6.896	5.058	30
Bicarbonate	1.00–95.00	13.94	23.74	0.3
Sulphate	1.30–88.0	17.69	23.04	250
Chloride	1.50–50.0	7.923	9.514	250
Nitrate-Nitrogen	0.013–9.72	2.224	3.330	10
Nitrite-Nitrogen	0.001–0.220	0.0220	0.049	3
E. Cond	1.140–97.80	26.91	30.088	
Alkalinity	4.80–558	141.81	157.026	
Hardness	0.001–9.50	1.789	2.576	
Phosphate	0.001–9.50	1.586	2.645	
TSS	1.0–4.500	1.323	0.893	

Pearson product correlation moment at *p* < 0.05 or *p* < 0.01 two-tailed revealed that there was no significant relationship between pH and major ions such as Na, Mg, K, Ca and SO_4_ in water samples from the Tarkwa mining area (refer to Table 4 below). However, a positive significant relationship existed between TSS and Turbidity, K and SO_4_, as well as between Mg and SO_4_ at *p* < 0.01 significant level two-tailed.

The correlation matrix in Table 5 below shows significant inter-metal relationships (*p* < 0.05 and *p* < 0.01). The Cd-Mn correlation is recognized as the weakest, with a correlation coefficient *r* = 0.446. Significant strong correlations (*r* > 0.5) were found between As-Cd, Pb-As, Pb-Cd, Pb-Mn, As-Zn, Mn-Cd, Hg-Mn and two more toxic metals, Hg-Pb. To explain the differences in correlations between the trace metals in each of the compartments, physical, chemical and biological processes occurring permanently in an aquatic environment (internal processes) as well as discharging of pollutants and other anthropogenic activities (external processes) and their effects on the partitioning and behaviour of heavy metals in that aquatic system must be taken into consideration.

**Figure 3:**
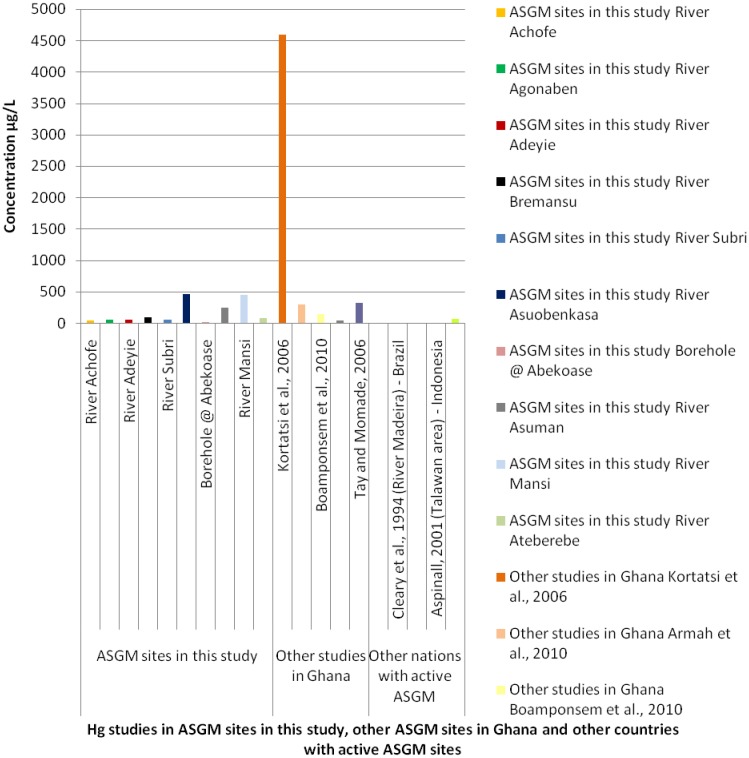
A graph comparing Hg levels in water samples from ASGM sites in this study with other ASGM sites in Ghana as well as other countries with active ASGM sites [6,28,29,30,32,33,34,35,36,37].

Other studies by different scientists have found a high degree of variability in concentrations of As, Hg, Mn, Cd and Pb in surface water from rivers or streams sediments in certain mining communities in Ghana. For example, the highest As concentration (3137 μg·L^−1^) was reported by [29,30]. Also, [31] reported the highest mean concentration of Hg (4600 µg·L^−1^) in water samples from the Tarkwa mining area. A comparison of Hg levels in water samples from this study with Hg levels from ASGM sites in other countries has revealed that Hg levels reported in the water are amongst the highest worldwide (see Figure 3 below).

**Table 4:** Pearson’s Product-Moment Correlation Coefficients for major ions in water samples from the study area.

	pH	Col	Turb.	TSS	TDS	Cond.	Alkalinity	Na	Mg	Ca	K	SO_4_
pH	1											
Col	−0.176	1										
Turb	0.065	**−0.267** *	1									
TSS	0.024	−0.113	**0.352** **	1								
TDS	0.161	0.095	−0.029	0.175	1							
Cond	0.176	−0.234	−0.038	0.047	0.147	1						
Alkalinity	−0.018	0.116	−0.104	0.071	0.040	−0.181	1					
Na	0.017	0.156	−0.033	0.098	−0.037	−0.126	0.047	1				
Mg	−0.034	0.065	−0.140	−0.128	−0.006	−0.158	**−0.293** *	0.231	1			
Ca	0.058	0.057	−0.146	0.241	0.037	0.142	**0.480** **	0.205	**0.386** **	1		
K	0.043	−0.067	**0.420** **	**0.575** **	0.119	0.016	0.055	0.064	0.058	0.091	1	
SO_4_	0.139	−0.055	**0.386** **	**0.564** **	−0.015	−0.070	−0.001	0.145	**0.349** **	0.168	**0.650** **	1

* Correlation is significant at the 0.05 level (2-tailed); ** Correlation is significant at the 0.01 level (2-tailed).

The correlation analysis was conducted to determine the relationship between metal concentration in water and the influence of physico-chemical parameters of water on metal concentrations. The pH and TDS are a major concern in this study since they are the vital factors in metal solubility and control metals speciation and thus their distribution within dissolved fractions [38]. In this study, concentrations of As and Cd were found to be negatively correlated (Table 5). According to Mackie [38] and Elzahabi and Yong [39], the solubility of heavy metals in water normally increases under acidic condition (pH *<* 4). However, the influence of pH on metal solubility is not obvious as the pH values in the present study ranged from 5.59 to 7.45.

Strong As-Cd, As-Pb, As-Mn, As-Hg metal-metal correlations suggest rivers in the Tarkwa mining area are highly polluted with mining effluents. Several factors might have caused this increase; notably among them are most of the ASGM activities such as washing of ores and amalgamation of gold ores with mercury takes place at these sites, remaining inputs are from improper disposal of mine tailings from the tailings dam via the emergency spill way [40].

**Table 5:** Pearson Product-Moment Correlation Coefficients between water quality parameters in Tarkwa Nsuaem Municipality, Ghana (*n* = 200).

	pH	Turb.	TDS	Cond.	As	Cd	Co	Mn	Pb	Hg
pH	1									
Turb	−0.237 **	1								
TDS	0.400 **	−0.169	1							
Cond	0.415 **	−0.182	0.993 **	1						
As	−0.367 **	0.146	−0.316 **	−0.367 **	1					
Cd	−0.534 **	0.037	−0.357 **	−0.373 **	0.664 **	1				
Co	−0.280	−0.038	0.020	0.016	0.079	0.614 **	1			
Mn	0.038	0.016	−0.045	−0.040	0.625 *	0.446 **	−0.122	1		
Pb	0.091	−0.061	−0.057	−0.033	0.829 *	0.603 *	−0.134	0.703 *	1	
Hg	0.035	−0.014	−0.0156	−0.128	0.701 **	−0.003	0.004	0.553 *	0.634 *	1

* Correlation is significant at the 0.05 level (2-tailed); ** Correlation is significant at the 0.01 level (2-tailed).

### 3.2. Socio-Economic Study

The socio-economic study focused on assessing the perceptions of residents of water quality in their area due to gold mining. Answers to the questions asked during the socioeconomic survey have been presented in Table 1 above. Of the interviewed families, 59% were males and the remaining percentage females.

Education is a key determinant of household formation, structure, socio-economic status and value judgment in every endeavor. The few residents with tertiary education (8.4%) as compared to the national figure of 40% in Tarkwa mining area means that they would have limited employment opportunities with mining companies operating within these areas. This is due to the fact that mining operations require a highly skilled labor force [40]. The resulting effect of this is the upsurge in the artisanal small-scale mining which requires no skills. Hence from Table 1 above, 45% of respondents in the study area have no formal education, 29% have their highest educational level to be Junior High School, only 3.4% have attained university education to the bachelors’ level, 5% having diploma, and the remaining 18% have their highest education to the senior school level [15,29].

Out of the 250 residents interviewed in the study area, 83.5% of them said their levels of economic activities, which mainly involved farming, were encouraging before the advent of mining activities while 10.5% had a contrary view. Of the 8% of the respondents in the study area who hold either a diploma or first degree from the university, only 2% are employed by the mining companies. This is because most mining companies employ large numbers of expatriate staff to fill positions which could be occupied by Ghanaians. This observation is consistent with studies made by [41,42].

The socio-economic dependencies of people in the study area on the mining industry is evident in the fact that 15% of the respondents had relatives who are mine workers; out of the 15% of the respondents who claim they had relatives’ employed by the mining industry; 10% of these mine workers have been laid off by the mining industries they have resorted to illegal mining as a way of meeting their livelihood needs.

For 83.5% of the respondents, farming was their main source of occupation before they ceded their lands to mining companies. This had resulted in a high unemployment rate of 65%, which should be considered as the main cause of many social problems affecting the livelihood of the people.

The majority of the respondents (90%) have some environmental concerns regarding gold mining activities in the study area. When the respondents were asked to be specific with their environmental concerns, 93% of the respondents said mining has polluted the water bodies in their communities. It was significant to note that 82% of the respondents could not drink from their traditional sources of water due to pollution (Table 1 above). 87% of the respondents said they have lost their farmlands as a result of expanding mining activities in the region. This can have a significant impact on livelihoods, particularly in Tarkwa where the average daily wage is less than $1 U.S. per day.

A stepwise logistic regression was used to estimate explanatory factors influencing the perception of residents on the socio-economic effects of mining in the study area. The responses to questions asked in the survey as well as the categories to which the question belong in logistic regression analysis have been summarized in Table 6 below.

**Table 6:** Results of the logistic regression analysis.

Perception of Water Quality (Model)	Predictors	Category	Β	*p*-Value	Odds Ratios
Highly polluted water bodies	Education	No formal education	3.12	1.21	4.46
		Junior High School	0.92	0.14	2.51
		Senior High School	0.78	0.14	2.17
		Diploma	0.23	0.72	1.25
		University degree	0.04	0.94	1.25
	Household income	Less than $1 dollar a day *vs* more	1.82	0.16	2.17
	Taste of drinking water	Deteriorated water quality	1.93	0.02	6.92
	Familiarity environmental problem	If mining activities continues, water bodies would be polluted	1.63	0.010	5.97
Stoppage of surface mining improves livelihood of residents of mining communities	Education	No formal education	1.33	0.052	3.79
		Junior High School	1.18	0.009	3.25
		Senior High School	0.04	0.840	1.04
		Diploma	0.03	0.941	1.01
		University degree	0.04	0.840	1.04
	Household income	Less than $1 a day vrs more	1.63	0.003	5.11

The results of the stepwise logistic regression revealed that, for the perception “highly polluted water bodies”, three predictors were most significant in explaining the opinion of residents of the study area interviewed in this survey (Table 6). These three predictors are education, household income and familiarity with environmental problems. Out of the five education-level groups, those with no formal education were about six times more likely to consider water bodies in the Tarkwa municipality as highly polluted than the Junior and Senior High graduates taken as reference category. This result is not consistent with the assertion that the more highly educated people are more likely to show concern to environment issues [41]. The second predictor was household income levels. The results showed that people living on less than 1 $ a day were more concern with their environment than those who live above this mark. This observation is consistent with the findings of [41]. The third predictor was familiarity with environmental problems associated with mining.

The few respondents who believed that the water quality had stayed the same or deteriorated compared to the period before the commencement of mining activity in the study area were six times more likely to perceive the water as being highly polluted than those who said that the quality water had improved since mining operation took place in the area. From Table 1 above, 68% of people in the study area are of the view that, if mining activities of ASGM should stop or are properly regulated by the agencies mandated by law, pollution of water bodies in the area would cease. That is, making heavy metal contamination of surface waters in Tarkwa a serious issue which requires an urgent attention [43]. It is also observed in Table 1 above that 83% of the respondents observed that the taste of drinking water in the study area was bad, and as such classified as highly polluted. The outcome of the perception study agrees with the chemical data in Table 2 above, indicating strong pollution levels of water bodies in the study area. There was a highly significant correlation between the three predictors and the perception model.

## 4. Conclusions

This study evaluated the impact of gold mining in Tarkwa mining area from both natural science and socioeconomic perspectives. The natural science study results revealed that pollution of water bodies in the area was attributable to uncontrolled cyanide spillages and acid mine drainage. Levels of arsenic, manganese, lead, cadmium and mercury in most cases exceeded GS 175-1/World Health Organization (WHO) permissible guideline values. These results agreed with the observations resulting from the socioeconomic survey. It was obvious from both the natural and social science studies that residents of the Tarkwa mining area perceived water bodies to be highly polluted due to mining. A significant finding from the study was that the people’s perception of the pollution of their environment was not directly linked to the level of their education. Though, mining is perceived to improve the general economic well-being of residents, the economic situation of the people of Tarkwa mining area is different as surface mining has deprived them of means of improving their livelihood through farming.
